# The complementary role of affect-based and cognitive heuristics to make decisions under conditions of ambivalence and complexity

**DOI:** 10.1371/journal.pone.0206724

**Published:** 2018-11-09

**Authors:** Carlos Andres Trujillo

**Affiliations:** Universidad de los Andes – School of Management, Bogota, Colombia; Universidad Loyola Andalucia, SPAIN

## Abstract

Little is known about the interplay between affective and cognitive processes of decision making within the bounded rationality perspective, in particular for the debate on adaptive decision making and strategy selection. This gap in the knowledge is particularly important as affect and deliberation may direct preferences in opposite directions. How do decision makers solve such dissonance? In this paper, we address this question by exploring the use of integral affect as a choice heuristic in comparison with and in conjunction to “take the best,” and weighted addition of attributes (WADD). We operationalize theories of reliance on affect in choice through a "Take the emotionally best" algorithm. Its predictive power is experimentally tested against other models, including mixed-sequential cognitive/affective procedures. We find that individual decisions are better predicted by a sequential combination of "Take the emotionally best" and "Take the best" with a slight dominance of the former. Conditions of cognitive/affective ambivalence, low discrimination ability and high complexity provide the cognitive architecture where such blended choice strategies predict decisions more precisely. This implies that reliance on integral affect may precede the use of cognitive cues following an ecological rationality perspective rather than supporting a kind of competition between affect and cognition as implied in current literature.

## Introduction

When making decisions, humans are motivated by what they *feel* and what they *think* is the best action. Without conscious control, our thoughts and feelings usually rush to our service and often compete for our attention. In its rational aspiration, western culture has promoted the education of reason to dominate emotion but research has shown that such a dynamic is hard to sustain. From the cognitive perspective, it is currently accepted that human beings are able to adjust the depth and extent of their reasoning process according to tasks and situational demands [1] (See a recent review and reflection on adaptive decision making and strategy selection in [2]. In particular, the studies of boundedly rational decision algorithms, better known as fast and frugal heuristics [3] [4], have operationalized the notion of ecological rationality through simple decision algorithms that operate as a toolbox for decision makers. Such algorithms are selected, mostly unconsciously, depending on how well they fit certain types of environments. Others have stressed that adaptation may also be achieved through a single-mechanism decision model that is used for all decisions but has certain components, such as decision boundaries and free parameters, that allow adaptation [2].

From the affective perspective, there is abundant research on the role of emotions in decision making. A number of works place particular emphasis on the complex interplay of reason and affect as a determinant of apparently rational decision making [5] [6] [7]. The dance of reason and affect is connected to various dual-process approaches such as System I and System II [8] [9] or impulsive and reflective [10] and it is recognized that their interplay may yield conflicting preferences [11] [12] [13] [14]. These works offer insights on how choice processes that are not deemed materially rational are, in fact, descriptive of human behavior and entail evolutionary value [6]. Little is known, however, about the way in which affective processing interacts or participates in boundedly rational processes of decision making. This gap in knowledge has already been identified [15] but apart from Lowenstein et al.´s [14] theoretical model of the interplay between affect and deliberation, there is no account of the affective aspect of bounded rationality and there is no empirical work exploring the topic. In particular, affect-based heuristics and cognitive-based heuristics may produce dissonant preferences and current knowledge has no answer regarding how decision makers solve this conflict or cognitive/affective ambivalence. This paper addresses this question. Current knowledge offers explanations on when reliance on affect may determine preferences. It is known, for instance, that risk may be evaluated from its affective component [16] and uncertainty increases reliance on affect [17]. Feelings are expected to be used to form judgments when they are perceived as relevant sources of information and when feelings simplify demanding tasks [18] [19]. We incorporate these principles of reliance on affect into a research design that captures heuristic affective processing through a choice procedure we call, Take the emotionally best (TTEB).

Through the proposed algorithm, we also explore how affect is processed in choice, contributing to further understand the role of affect in decision making [20] [21]. The few works that address affective processing in choice are very contextual, such as choosing political candidates [22] and risk attitudes [13]. To develop a more general account of affective processing in choice, following theories on dual systems of thought, bounded rationality and reliance on affect to form judgments, we align our methods to previous research on choice strategies and algorithms [23] strategy selection [2], and to studies on how bounded rationality is operationalized through fast and frugal heuristics [3] [24], which are very detailed in terms of how information is processed. In short, this paper helps us understand how humans solve the ambivalence between affective and deliberate processing [14] within the bounded rationality perspective, and shed new light on the conditions under which people rely on affective responses to make decisions. We adopt an architectural perspective by studying three complementary aspects of the predicting ability of the TTEB algorithm: discrimination ability, task complexity and strategy redundancy. We discuss the results of the comparative performance of TTEB in light of theories of multi-mechanism and single-mechanism strategy selection, reliance on affect and constructive decision processes.

## Background

Several approaches have been used to study emotions and affective processing in decision making. Emotions could be anticipatory [25], meta-cognitive [26], and they can take the form of discrete feelings (e.g., regret, guilt, etc.) [27] whose associated action tendencies interact with rational thinking. Peters [28] explains four functions of affect in the construction of preferences, namely, affect as information, as a spotlight for different types of information, as a motivator to action, and as common currency to compare different types of information. We focus on integral affect from an affect-as-information perspective, as the key emotional element that fits into a boundedly rational decision process. Integral affect is the emotional response that is elicited by features of the decision target: real, perceived, or imagined [21]. This type of affect may play a salient role in decision making processes because it is used as a resource-efficient proxy for value [6], which implies that people infer information about decision objects from the affective responses such objects elicit [19]. The resource-efficient and informational value of integral affect lead to increased reliance on this when facing constrains in processing, mostly attributable to cognitive load [26] [29]. Integral affect tends to be holistic and insensitive to scale, magnitude [30], and probabilities [16], and it is highly accessible [31]. Such characteristics lead us to expect that judgments and choices based on Integral Affect are mostly connected to associative based decision making. Associative and rule-based systems of thought [8] engage different computational processes. The former, also known as System I, appeals to automatic, non-conscious processing that most likely requires long term associations. The latter, on the other hand, also known as System II appeals to deliberate, conscious processing that tends to exhaust short-term memory. [8] This is not to say that System I is entirely emotional, but that integral affect, from its informational nature, is a salient constituent of intuition.

We argue that integral affective responses, being holistic and directly triggered by decision alternatives, constitute an adequate emotional dimension to be studied from an adaptive information-processing framework [1]. Other perspectives of affect on decision making speak of predispositional and contextual influences and sometimes make certain emotional states objectives in themselves (e.g., minimizing regret).

### The interplay between affect and reason in decision making

The interaction of emotions and reason in decision making is a problem that tackles the core of human behavior and has been studied from multiple angles. In this research, we focus on the frequent situation when humans encounter choices in which a reason-based evaluation of options suggests a course of action that differs from more intuitive and emotional impulses. Simply put, people may be subject to conflicting thoughts and feelings when making decisions. Such inconsistency may be attributable to humans operating under dual-process models of thought [9]. Theoretical models of such interplay take into account the potential contradictory motives of the two systems [14]. As mentioned above, the representation of these thinking modes as System I and System II [31] [32] is currently widely used. Others have referred to impulsive and reflective processes [10] or hot and cold modes of thought [33]. While making choices, the two processes are operating in parallel [8] and given their different nature, they may point in different directions. It has been proposed that dissonant or ambivalent affective-cognitive structures tend to favor reliance on affect in problem solving [34] and attitude formation [35]. Such potential ambivalence is notoriously understudied in behavioral decision theory. In this paper, we address that gap by taking these ideas to the realm of choice heuristics in order to empirically explore the role of integral affective responses towards choice alternatives in comparison to well-known cognitive choice algorithms. We will attempt to assess the magnitude of the cognitive/affective ambivalence and study the way in which both affective and cognitive choice algorithms perform under such conditions.

### Fast and frugal heuristics, strategy selection and take the emotionally best

Fast and frugal heuristics are boundedly rational and adaptive (ecologically rational) choice algorithms [3] that serve as a multi-mechanism toolbox for the decision maker. Simply put, this means that these algorithms are available to enable the decision maker to arrive at sufficiently good decisions through choice algorithms that are suitable for the environment in which they are embedded. In contrast, materially rational, utility maximizing models such as linear regression or multiattribute utility theory [36] are expected to be independent of the environment, hence they are not adaptive. More recently, a new set of single-mechanism models have been introduced that may achieve adaptation through free parameters and other model elements, such as evidence accumulation and stopping rules (See [37] for a recent review). Furthermore, the fast and frugal heuristic toolbox is currently challenged in its traditional view, as new methods and theories explore the advantages of blending strategies [38], while others study the performance of simple heuristics when relaxing the assumption of independent dimensions [39]. Saliently, affect—based choice is absent from these discussions.

If we consider the emotionally best (TTEB), the choice procedure tested in this paper contributes to addressing that open question. TTEB consists of choosing the alternative that integrally triggers the most positive holistic affective response. TTEB is based on the notion of the affect heuristic developed by Slovic et al. [32] and feelings as information theory [40]. They argue that to form judgments and to construct preferences, individuals look at the affective valence of decision alternatives and use this as a source of valid information. Reliance on how alternatives feel in order to guide preferences is using an “affect heuristic.” Hence, TTEB directs the individual to choose an alternative that feels good, mostly driven by environmental and task conditions when reliance on affect is ecologically convenient. We argue that there are at least three of these conditions. The first of these occurs when there is cognitive/affective ambiguity, that is, cognitive and affective information point to conflicting preferences; under the second condition, discrimination ability of cognitive strategies is low; and finally, the third condition refers to when task complexity is high. These conditions can be considered part of the cognitive architecture in which the decision maker operates. In this paper, we explore the extent and the conditions under which integral affective responses towards decision alternatives are used in comparison to, and in conjunction with, cognitive-laden choice strategies. Our approach enables an exploration of whether TTEB can be considered one of the available strategies in the heuristic toolbox, or whether it works better under a blending of strategies.

## Method

This research was approved by Universidad de los Andes vice presidency of research and by the School of Management research committee. As part of this approval, the school of management research committee reviews compliance with ethical standards and request authors to course and approve the CITI program on ethics in research. Within the study protocol, informed consent was obtain by explaining to participants the experiment in the first screen of the computer based experimental platform, on which they were sked to agree to voluntary participate.

Information processing must be observed, in order to test the hypotheses. To do so, the two main available techniques are a) looking at the process through verbal protocols or people´s information search, and b) looking at the outcomes by carrying out a comparative fit of models using actual data [41] [24]. In this work, the second approach was taken. The rationale of setting a model competition is that, first, affective processes are hard to observe through verbal protocols. Doing so entails asking people to reflect on their affective reactions in order to transform primary affective responses into self-reported discrete feelings (surprise, anger, elation, etc.) that are subject to individual interpretations. Second, integral affect has been linked to somatic markers [6] [42]. These are emotional responses generated by the recall of a personal or hypothetical emotional event that is associated to a current experience, and that elicits a somatic state when brought to working memory, for example, when evaluating decision alternatives. Accumulated empirical evidence on this phenomenon points to the fact that the origin of such learned responses is the ventromedial prefrontal cortex in connection to the amygdala [43]. This cognitive-affective link is, however, very complex, as it involves multiple overlapping brain networks in both cortical and subcortical regions that are dynamic and context sensitive [44]. It would therefore be very difficult to use neuroimaging or other techniques to trace the specific dynamic -let alone content- of affect-as-information processing implied by integral affective responses, and hence, impractical for this research. Thus, an experiment was conducted in which people were asked to make choices about consumer products. Four choice models were fitted to the data to examine which was better able to predict the participants’ actual choices.

In order to activate the adaptive mechanisms that trigger the use different choice algorithms, the experimental manipulation is carried out through intrinsic cognitive load[45] and component complexity [46] by altering the number of elementary information processes or information cues [1] [47]. Two treatments are defined:

1. Low complexity: Choices between two alternatives with two attributes each
2. High complexity: Choices among four alternatives with four attributes each

Cognitive load is the information-processing burden that is required for the working memory to perform a given task, in this case, a choice task. It is a well-established fact that working memory is very limited and the number of information items that can be handled is very small. The exact number is a matter of academic debate, but it is most likely less than ten, even after "chunking" [48] [23]. In this study, the low complexity treatment implies the handling of at least four items of information, while high complexity implies the handling of at least 16 items. Such manipulation is also consistent with the notion of task component complexity [46]] according to which, the number of information cues required to complete a task, monotonically increase its complexity. In addition, choice sets are designed so that there are no dominant alternatives; hence, implicit trade-offs contribute further to increasing cognitive load and complexity. Complexity is also expected to trigger the use of heuristic strategies [49] [23].

### The competing models

#### Weighted additive rule (WADD) [23]

This choice procedure takes into account all the available information about the decision options. All available alternatives’ attributes *x*_*i*_ are multiplied by their importance (weight) *w*_*i*_ to obtain a total value or utility for alternative *j*. This is valid from a normative standpoint as it allows utility maximization by comparing the available alternatives. Thus, if *U*(*A*) > *U*(*B*), then A is chosen. This choice procedure can be described by the expression:

$$U(j)=\sum_{i=1}^{n}x_{ij}w_{ij}$$ (1)

where *i* ∈ {1, ‥, *n*} attributes, which are specific to Alternative *j*. This specification assumes that values of *x* are observable. However, some attribute values are not easily measured (e.g., quality, attractiveness, usefulness). It is therefore assumed that the decision maker is able to transform any relevant attribute information into ordinal attribute utilities and Eq 1 becomes the more general expression

$$U(j)=\sum_{i=1}^{n}u(x_{ij})w_{ij}$$ (2)

#### Take the best (TTB) [50]

As explained earlier, TTB basically consists in choosing the alternative that is considered the best in the most important attribute, disregarding other attributes. Only if two or more alternatives turn out to be equally good in the most important attribute, the second best attribute is used, using what is called a lexicographic procedure [1].

#### Take the emotionally best (TTEB)

This choice procedure consists of choosing the alternative that triggers the most positive integral affective response, taken as the holistic emotional effect triggered by the alternative, not by specific features or attributes of the alternatives. This is derived from the notion of the affect heuristic proposed by Slovic et al. [32] who show across different domains of judgment and decision making that people experience the "goodness" or "badness" of stimuli as a feeling state. Moreover, they show that reliance on such experiences explains several well-documented decision making phenomena (e.g., evaluability [51] such as risk evaluation [52] among others. TTEB is a specific operationalization of the affect heuristic as a choice algorithm where the experience of integral affective responses guides preference.

#### TTB then TTEB

This is a sequential choice procedure that assumes that cognitive processing somehow precedes the use of emotional considerations (i.e., affect-as-information). The choice maker starts by using TTB and the integral and holistic affective response would be used only when TTB is not able to discriminate between alternatives. This is not to say that cognitive processing actually happens faster than affective processing. Rather, it means that people deliberately focus on the cognitive procedure (TTB) before letting their integral emotions guide their preferences.

#### TTEB then TTB

It is also considered that a sequential affective/cognitive choice procedure may happen in the opposite order. That is, people start by focusing on the integral emotions to guide the initial preferences, and only when these responses are not clear enough to discriminate between alternatives, do they engage in the cognitive processing of information through TTB.

These two sequential choice procedures are likely to happen if, as explained earlier, there is some degree of cognitive/affective ambivalence between what people feel and think about alternatives. People are not fully aware of the origin of their emotions and therefore there may be features of the choice alternatives that trigger integral affective responses through unconscious processes. That would engender contradictory preferences from WADD, TTB and TTEB choice processes which people must resolve. The two cognitive/affective sequential choice procedures are of special interest because they may solve ambivalence originated in boundedly rational reasoning.

### Experiment

#### Participants

The experiment was conducted at a major private university where students were recruited through campus announcements. Ninety-eight undergraduate students participated, 51 of whom were randomly assigned to the high cognitive load treatment, and 47 to the low cognitive load treatment. The whole sample generated a panel of 490 choices. Ages ranged from 18 to 23 (Mean = 20; SD = 1,31) and 55% were men. They received a flat fee of 7 USD for participating, which is the approximate hourly opportunity cost of an undergraduate student in the geographic area where the experiment took place. Before starting the experiment, they were informed that participation was voluntary and there were no risks involved. All chose to participate. The procedures were in accordance with the University’s ethical standards.

#### Procedure

People were asked to make choices regarding high involvement consumer products: Laptops, cars, restaurants, digital cameras, and mobile phones. High involvement is convenient because involvement leads people to engage in lengthy reasoning and process more information [49] [53]. This condition offers all competing choice procedures a real probability of happening. Participants were randomly assigned to high or low complexity conditions. We chose to use a between subjects design to avoid the potential problem of false consistency among choices and common methods bias [63] [54] if a within subjects design were implemented. Participants had to make five choices, one per each product category. For example, a participant in the low complexity condition would make a choice between two cars, then between two laptops, and so forth. The order of choices and the attributes were fully randomized and counterbalanced using the computer platform where the experiment was programmed. We selected product attributes that were highly independent. For instance, in cars, fuel consumption and warranty are reasonably independent. The goals of this are twofold: First, independent attributes increase trade-off difficulty and second, attribute dependence or independence affects the performance of TTB and WADD as the two models are expected to satisfy the principle of dimension independence (See [39]). S1 Table contains the choice alternatives and their attributes. The experiment was conducted on computers and programmed on blackboard software.

Once the choice task was completed, people were asked to state their affective responses to the alternatives, one alternative at a time and non-comparatively. Alternatives and attributes were again randomly presented to participants sequentially. Integral affective responses were captured using the Self Assessment Manikin (S.A.M), a measurement tool for emotions that uses facial recognition to elicit responses in the Pleasure, Arousal, Dominance (PAD) emotional spaces, capturing a wide range of feelings [55]. The Manikin reliably captures underlying appetitive and aversive motivational forces of emotional reactions [56]. This type of self-reported measure works for this study, as it allows the experimenter to direct participants towards specific stimuli, capturing integral affective responses towards those stimuli. In addition, facial recognition of emotions reduces variations in the subjective interpretation of feelings [57] [58]. See S1 Fig for a sample of the S.A.M instrument.

Finally, attribute weights were obtained by asking participants to distribute 100 points among attributes. This time, the alternatives were not presented to participants. Instead, a more general question was used such as: “when choosing a laptop, how important are the following attributes (i,‥,n)?, please distribute 100 units among those attributes in order to answer the question.” Participants in the high cognitive load condition weighed the four attributes of each product and participants in the low cognitive load condition weighed two. Products and attributes were presented randomly. Finally, consumer involvement with each product was measured using the revised PII (personal involvement inventory) [53].

## Results

### Descriptive

#### Preferences

In the low complexity group (2 choices, 2 alternatives each), 58% chose Alternative A, and 42% Alternative B. In the high complexity group (4 choices, 4 alternatives each), 24% chose A, 34% chose B, 33% chose C, and 9% chose D. The order of presentation of alternatives and attributes were fully randomized through the computer platform. This result means that there was ample variance among choices as preferences were distributed across all options in both treatments.

#### Integral affect

Affective responses captured by S.A.M were coded only for the pleasure dimension, as it is this that captures the directionality of the emotion, which, in turn, provides the individual with clear information about preferences. The other dimensions, namely arousal and control, have no clear theoretical interpretation in terms of affect-as-information in connection to preferences. S.A.M. uses a 9-point scale for each dimension. Pleasure (also called valence) was coded from -4 to 5, omitting 0 in order to capture the directionality of the response. In the high complexity treatment, mean valence was 1.13, 1.02, 1.06, and 0.20 for alternatives A, B, C and D respectively. A multivariate test of means reveals significant differences (F = 12.4; p < 0.01), which are caused by the lower value of valence for Alternative D. In the low complexity treatment, mean valence was 1.57 and 1.21 for alternatives A and B respectively. This difference is significant as revealed by a t-test for paired variables (t = 2.59; p < 0.05). Arousal was coded from 1 to 5, with intervals of 0.5 to capture the 9 possible values of S.A.M. Mean arousal was 2.9, 3, 2.88 and 2.77 for alternatives A, B, C and D respectively. No significant differences were found, as revealed by pair wise t-tests. We also compared differences in arousal between treatment groups: For Alternative 1, there was no difference and for Alternative 2 arousal was slightly higher for the low complexity group (mean _high complexity_ = 2,86, mean _low complexity_ = 3,14; t = 2,38; p = .02).

#### Attribute weights

**Fig 1:**
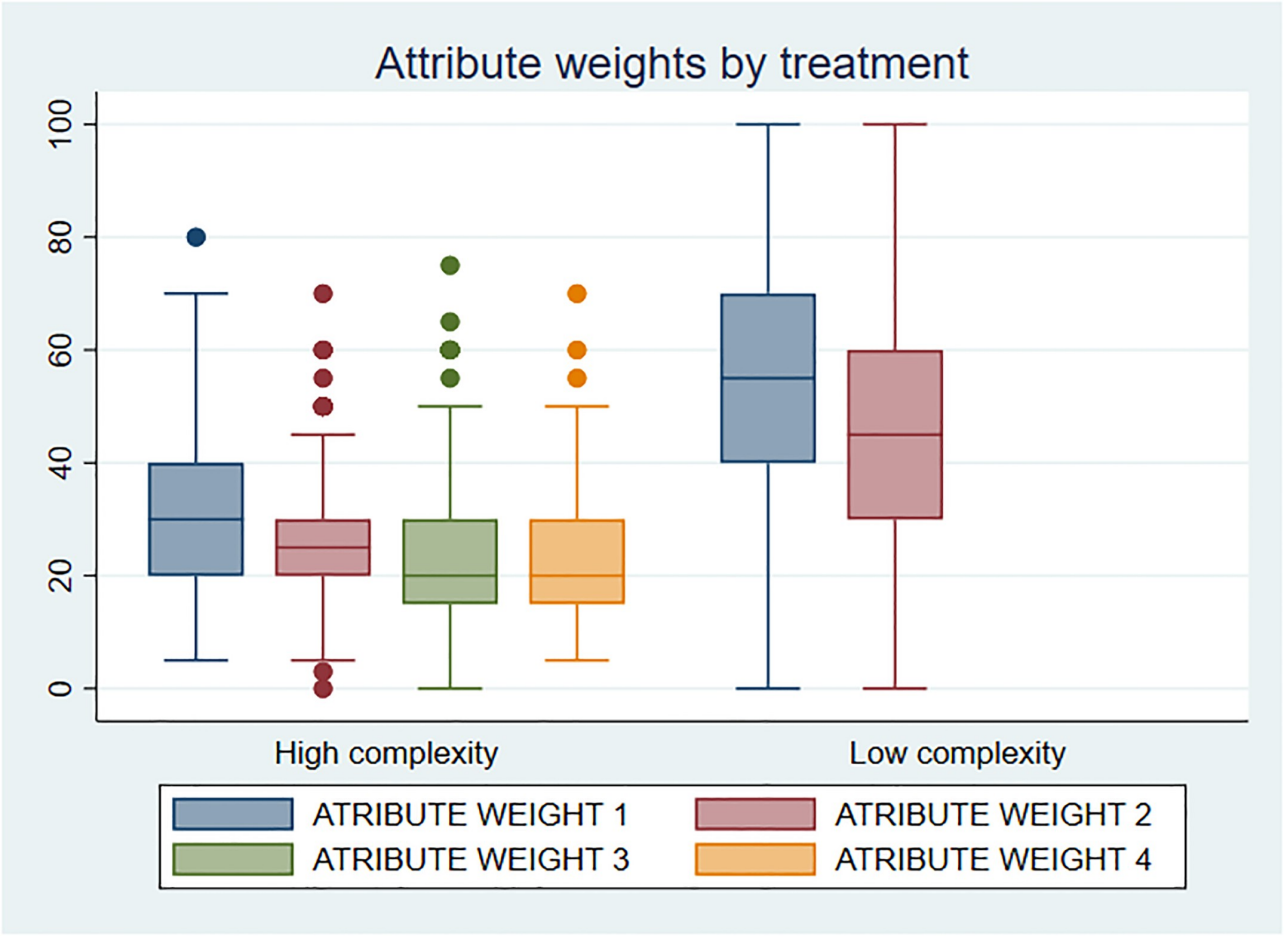
Attribute weights by treatment.

The mean weight of each attribute, classified by product, is reported in Table 1. Although there are statistical differences among attributes in most products, there is no evidence of dominant or overweighed attributes that may have directed choice towards particular alternatives. In the high complexity treatment, the mean weight for attribute one was 30.7 (SD = 13.5); for attribute two, it was 24.7 (SD = 11.3); for attribute three, it was 22.3 (SD = 13.2); and for attribute four, it was 22.2 (SD = 10.5). Some differences were statistically significant, mostly attribute one over the others: (t [1 vs. 2] = 4.24; p .00; t [1 vs. 3] = 5.3; p = .00; t [1 vs. 4] = 6.64; p = .00; t [2 vs. 3] = 1.86; p = .06; t [3 vs. 4] = .09; p = .9). However, the largest difference (Attribute 1 over Attribute 4) was only 8 points out 100 possible. In the low complexity treatment, in which only two attributes were used, the weight of attribute one was 53.8 (SD = 21.2) and 45.5 (SD = 21.1) for attribute two (t = 3.16, p 00). There was heterogeneity among participants in the weights they assigned to attributes, meaning that the research design allowed the collection of a counterbalanced sample of choices. Fig 1 contains boxplots showing the distributions of attribute weights by treatment. Heterogeneity in attribute weighing is reflected in the wide range of values for all attributes regardless of treatment (from very low to very high) and the dispersion of values.

**Table 1:** Average attribute weights by treatment.

**Laptop (High)**							**Laptop (Low)**						
**Attribute**	**N**	**Mean**	**Std. Dev**.	**Min**	**Max**	**Statistical Test**	**Attribute**	**N**	**Mean**	**Std. Dev**.	**Min**	**Max**	**Statistical Test**
Screen size	43	25.23	11.14	5	50	ns	Screen size	51	40.63	13.34	13	75	t = 25.16***
Memory	43	27.81	11.42	10	60		Memory	51	59.37	13.34	25	87	
Hard drive	43	24.81	9.05	5	50								
Battery	43	22.14	11.23	5	70								
**Car (High)**							**Car (Low)**						
**Attribute**	**N**	**Mean**	**Std. Dev**.	**Min**	**Max**	**Statistical Test**	**Attribute**	**N**	**Mean**	**Std. Dev**.	**Min**	**Max**	**Statistical Test**
Engine capacity	43	31.07	12.11	10	60	F = 12.74**	Engine capacity	50	59.32	19.98	0	95	t = 10.88 ***
Warranty	43	24.21	12.69	5	70		Warranty	50	40.68	19.98	5	100	
Fuel tank capacity	43	21.42	8.81	5	50								
0- 60m/h acceleration	43	23.30	10.51	10	60								
**Restaurant (High)**							**Restaurant (Low)**						
**Attribute**	**N**	**Mean**	**Std. Dev**.	**Min**	**Max**	**Statistical Test**	**Attribute**	**N**	**Mean**	**Std. Dev**.	**Min**	**Max**	**Statistical Test**
Waiting time to get a table	42	27.93	12.13	5	50	F = 41.92***	Waiting time to get a table	50	47.18	20.72	10	90	ns
Average meal price	42	23.93	11.84	5	60		Average meal price	50	52.82	20.72	10	90	
Ranking	42	30.71	18.77	5	75								
Commuting time	42	17.43	8.51	5	33								
**Cell Phone (High)**							**Cell Phone (Low)**						
**Attribute**	**N**	**Mean**	**Std. Dev**.	**Min**	**Max**	**Statistical Test**	**Attribute**	**N**	**Mean**	**Std. Dev**.	**Min**	**Max**	**Statistical Test**
Battery talking time	43	31.67	14.22	5	70	F = 15.00***	Battery talking time	50	60.02	23.35	10	100	t = 9.21***
Charging time	43	23.74	10.85	3	45		Charging time	50	39.98	23.35	0	90	
Memory	43	18.35	13.13	0	60								
Processor´s speed	43	26.21	10.58	10	55								
**Digital Camera (High)**							**Digital Camera (Low)**						
**Attribute**	**N**	**Mean**	**Std. Dev**.	**Min**	**Max**	**Statistical Test**	**Attribute**	**N**	**Mean**	**Std. Dev**.	**Min**	**Max**	**Statistical Test**
Picture Resolution	41	38.41	14.83	15	80	F = 61.61***	Picture Resolution	51	63.75	16.10	0	90	t = 37.16***
Zoom	41	24.07	9.69	0	50		Zoom	51	36.25	16.10	10	100	
Weight	41	16.00	7.92	5	40								
Screen size	41	21.49	9.79	5	50								

p < .01 *,

p < .05 **,

p < .001 *** (ns) non significant

#### Involvement

Consumer involvement is the perceived relevance of a product [49] [53]. Involvement increases effort; thus, it was measured in order to control for effort effects in choice. As expected, using the revised PII scale, the five products generated relatively high mean levels of involvement: 34.04 for laptops, 33.79 for cars, 31.83 for restaurants, 30.49 for cell phones and 29.29 for digital cameras. The maximum possible value for this scale is 42. These differences were statistically significant (F = 8.86 p < 0.01) but all products yielded involvement levels in the upper third of the scale. This allowed us to control for effort, avoiding such a confound factor when analyzing choice processes.

### Emotional and cognitive effects on choice probability

We analyzed whether integral affective reactions to alternatives and attribute weights were statistically associated to the probability of choice. For the low complexity treatment, we conducted random effects logistic regressions using the measure of the valence dimension to capture the integral affective response and the weights given to attributes to capture cognitive information. We controlled for involvement. Results are reported in Table 2. We found that the probability of choosing Alternative 2 (coded as 1) is positively and significantly associated to the affective response to Alternative 2 and negatively and significantly associated to the affective response to Alternative 1. Regarding weights, the probability of choosing Alternative 2 was negatively and significantly associated to the weight of Attribute 1. By design, Alternative 1 was superior in Attribute 1, and Alternative 2 was superior in Attribute 2; hence, this result is coherent with the experimental design. We conclude that both affective responses and cognitive information influenced the probability of choice.

**Table 2:** Random effects logistic regression of attribute weights and affective responses on probability of choice—Low complexity.

(a)	Coeff (S.E)	z	p value
SAM valence to alternative a	-.57 (.15)	-3.85	.00
SAM valence to alternative b	.93 (.16)	6.00	.00
Weight of attribute 1 (b)	-.03 (.01)	-2.95	.00
Involvement level	.01 (.03)	.54	.59
Constant	.17 (1.0)	.16	.87
N = 212, 49 groups			
Wald chi2 = 43.4 p = .00			

^(a)^Coding scheme: Alternative a = 0; alternative b = 1

^(b)^Weight of attribute 2 = 1 − weight of attribute 1, hence it is not reported for multicollinearity

For the high complexity condition, we estimated a multinomial logistic regression to account for the probability of choosing the 4 different alternatives. Results are reported in Table 3. We used Alternative 1 as the basis for comparison in the regression. Each of the table’s panels shows the results for each alternative. We find that the affective response to Alternative 1 is negatively and significantly associated to the probability of choosing all other alternatives. For alternatives 2, 3 and 4, the affective response to each of them is positively and significantly associated to its probability of being chosen. In contrast to the low complexity treatment, weights of attributes were not significantly associated to the probability of choice. We conclude that these initial analyses suggest that reliance on affect is observed in both conditions, whereas reliance on cognitive deliberation was only observed in the low complexity condition.

**Table 3:** Multinomial logistic regression of attribute weights and affective responses on probability of choice—High complexity.

(a)	Coeff (S.E)	z	p value
**Alternative b**			
SAM valence to alternative a	-.86 (.29)	-2.92	.00
SAM valence to alternative b	.82 (.24)	3.37	.00
SAM valence to alternative c	.18 (.16)	1.11	.26
SAM valence to alternative d	-.22 (.15)	-1.46	.14
Weight of attribute 1	.00 (.01)	.11	.91
Weight of attribute 2	-.00 (.01)	-.28	.77
Weight of attribute 3	.02 (.02)	.97	.33
Weight of attribute 4	-.00 (.02)	-.33	.73
Involvement	.00 (.03)	.22	.82
**Alternative c**			
SAM valence to alternative a	-1.45 (.32)	-4.49	.00
SAM valence to alternative b	-.31 (.23)	-1.39	.16
SAM valence to alternative c	1.17 (.25)	4.63	.00
SAM valence to alternative d	-.21 (.18)	-1.19	.23
Weight of attribute 1	.01 (.02)	.60	.54
Weight of attribute 2	.00 (.02)	.25	.80
Weight of attribute 3	.06 (.02)	2.42	.01
Weight of attribute 4	.01 (.02)	.45	.65
Involvement	-.04 (.04)	-1.00	.31
**Alternative d**			
SAM valence to alternative a	-1.05 (.37)	-2.86	.00
SAM valence to alternative b	-.03 (.30)	-.12	.90
SAM valence to alternative c	.17 (.28)	.61	.54
SAM valence to alternative d	.95 (.39)	2.41	.01
Weight of attribute 1	-.04 (.03)	-1.23	.22
Weight of attribute 2	.00 (.03)	.07	.94
Weight of attribute 3	.01 (.03)	.38	.70
Weight of attribute 4	.04 (.03)	1.55	.12
Involvement	-.04 (.05)	-.77	.43
N = 165			
Pseudo R2 = .38 p = .00			

^(a)^ Alternative a is the base outcome for comparison

### Comparison of model performance

The second stage of the analysis was to conduct a model "competition" to find out which of the previously explained algorithms of choice best-predicted actual choices. To do so, several steps were taken and a predicting procedure for each choice algorithm had to be established. The procedure included a calculation of discriminant ability; that is, the power of each model to discriminate between alternatives, offering the decision maker a clear preference. This is calculated as the percentage of choices for which the choice algorithm can discriminate.

First, to fit the WADD (Weighted additive) procedure, we used Eq 2: $U(j)=\sum_{i=1}^{n}u(x_{ij})w_{ij}$. We directly measured *w*_*ij*_ but there was a problem of scale comparability in the *x*_*ij*_ across the different products. To solve this, we transformed attribute values into ordinal utilities such that *u*(*x*_*ij*_) ∈ {.01, .25, .5, 1} for the high complexity treatment (where we defined 4 attribute levels) and *u*(*x*_*ij*_) ∈ {.5, 1} for the low complexity treatment (where we defined 2 attribute levels). Thus, we have measures of both *w*_*ij*_ and *u (x*_*ij*_*)* and can therefore calculate *U (j)* for all alternatives and assume that the DM chooses the one with the highest utility. If there is a tie in utilities between at least two alternatives, the model is unable to discriminate. For TTB (Take the best), we defined that the DM chooses the alternative that is best in terms of the most important attribute. If there is a tie between alternatives, we try to solve it using the next most important attribute, following a lexicographic procedure. For subjects that express equal weighting of attributes, the algorithm cannot discriminate between them. In the case of TTEB (Take the emotionally best), we used the answers to the SAM scale in the valence dimension. We defined that the DM chooses the alternative that triggered the most positive response in the measured -4 to 9 scale. If there is a tie among affective responses, the algorithm is unable to discriminate between them. For the two cognitive affective sequential heuristics (*TTB then TTEB*; *TTEB then TTB*), we simply applied them in the corresponding sequence for the decisions for which the first one was unable to discriminate. In the first case, if TTB could not discriminate, then TTEB was used; and, in the second case, if TTEB was unable to discriminate, then TTB was used.

We also evaluated the conditions for model comparison, which are necessary to evaluate the hypotheses. First, we checked overall predictability -the percentage of choices for which all models were able to discriminate-, which was 36% in the low complexity condition and 15% in the high complexity condition. These low percentages show that DMs need to adopt different choice approaches in order to make effective decisions. Put differently, if all models are able to discriminate a high percentage of decisions, the individuals may develop some preference for "how to choose" instead of changing the choice algorithm to fit the decision environment. This is consistent with theories of adaptive decision behavior [1] [2]. Second, we checked model redundancy; that is, the correlation of choice predictions among the algorithms. We found no significant associations in the high complexity condition whilst, in the low complexity condition, the only significant association was between WADD and TTEB (48%, p < .01), which may indicate that under low complexity affective, responses were somehow related to utility. However, when checking the presence of cognitive affective ambivalence; that is, the situations in which affective responses point in a different direction from the cognitive procedure (WADD or TTB), we found that under the high complexity condition, 91% of decisions were ambivalent and in low complexity, 76% of decisions were ambivalent. Further on in this article, we discuss the implication of these findings for dual-system theories and for the cognitive/affective ambivalence as a feature of the decision environment.

Finally, to evaluate the performance of the choice algorithms, we simply calculated the percentage of choices that each algorithm predicted correctly. We report three performance percentages: 1) overall performance, all choices included; 2) performance over decisions for which cognitive/affective ambivalence was observed; and 3) performance over decisions for which no cognitive/affective ambivalence was observed. This segmentation allowed us to analyze whether individuals follow affect or reason in the presence of contradicting and consistent preferences. Tables 4 and 5 (next page) contain the results by treatment.

**Table 4:** Comparative model performance: Low complexity choices, base rate of comparison: 50%.

Model	Performance	Discrimination	n total	z	P-Value
TTB					
Overall	59%	92%	252	1.99*	0.047
Over univalence	91%		56	9.82**	0
Over ambivalence	48%		177	-0.44 (ns)	0.66
TTEB					
Overall	79%	73%	255	6.21**	0
Over univalence	95%		45	10.11**	0
Over ambivalence	72%		142	4.65**	0
TTB then TTEB					
Overall	58%	96%	255	1.80 (ns)	0.073
Over univalence	92%		59	10.32**	0
Over ambivalence	48%		187	-0.45(ns)	0.654
TTEB then TTB					
Overall	75%	96%	255	5.77**	0
Over univalence	95%		59	11.23**	0
Over ambivalence	69%		187	4.33**	0
WADD					
Overall	87%	92%	252	8.71**	0
Over univalence	97%		56	11.59**	0
Over ambivalence	75%		177	5.67**	0

**Table 5:** Comparative model performance: High complexity choices, base rate of comparison: 25%.

Model	Performance	Discrimination	n total	z	P-Value
TTB					
Overall	33%	88%	212	1.76 (ns)	0.078
Over univalence	24%		19	-0.23 (ns)	0.817
Over ambivalence	34%		168	1.97*	0.049
TTEB					
Overall	63%	52%	235	7.03**	0
Over univalence	62%		12	6.86**	0
Over ambivalence	63%		111	7.03**	0
TTB then TTEB					
Overall	35%	90%	230	2.29*	0.022
Over univalence	24%		21	-0.24 (ns)	0.808
Over ambivalence	36%		187	2.5*	0.012
TTEB then TTB					
Overall	51%	89%	235	5.66**	0
Over univalence	62%		21	7.88**	0
Over ambivalence	50%		189	5.46**	0
WADD					
Overall	27%	74%	213	0.44 (ns)	0.664
Over univalence	10%		16	-3.67**	0
Over ambivalence	21%		142	-0.90 (ns)	0.367

The tables report performance measures, discrimination ability of the models and statistical tests of the difference between the performance percentages and a base rate of random performance (50% in the low complexity condition: 2 alternatives, and 25% in the high complexity condition: 4 alternatives).

#### Performance of WADD

In the low complexity condition, this model of choice has a very good level of discrimination (92%) and fits actual choices significantly better than random choice, mostly when there is cognitive/affective univalence. Its performance, however, decreases under cognitive/affective ambivalence (z = 3.60; p < .01) but it is still significantly better than random. In high complexity, the result is remarkably different. It does not fit choices significantly better than the 25% random base and, under cognitive/affective ambivalence, its performance is only 10%, which is significantly below random performance.

#### Performance of TTB (Take the best)

In the low complexity condition, TTB was able to discriminate alternatives as much as WADD which is consistent with current knowledge on the use of heuristics and notions such as the "less is more" effect [4]. TTB predicted actual choices very well when there was no cognitive/affective ambivalence. When affect and deliberation pointed in different directions, TTB did not perform better than random choice. Overall, TTB predicted actual choices marginally better than random. In the high complexity condition, TTB still displayed a good level of discrimination, but it fitted actual choices slightly better than random. However, in contrast to low complexity, the best performance was observed for choices where cognitive/affective ambivalence was present.

#### Performance of TTEB (Take the emotionally best)

In the low complexity, condition, the purely affective choice procedure had the lowest discrimination capacity (73%) but within that subset of decisions, it predicted actual choices remarkably better than random choice, overall and regardless of cognitive/affective equivalence or inconsistency. The best fit was observed for cognitive/affective equivalence. In the high complexity condition, complexity clearly affected its discriminant ability, but once again, where discrimination was possible, the heuristic predicted actual choices much better than random choice, and such performance was very similar for both cognitive/affective equivalence and ambivalence.

#### Performance of mixed-cognitive/affective sequential heuristics (TTB then TTEB; TTEB then TTB)

In the low complexity condition, both sequential heuristics achieved the highest discrimination ability (96%). The performance of both under cognitive/affective equivalence was very good, although *TTEB then TTB* did marginally better. Under ambivalence, *TTEB then TTB* was superior (69% to 48%; z = 4.12: p < .001). In fact *TTB then TTEB* did not fit actual choices better than random at all for cognitive/affective ambivalent decisions, and did so marginally overall. In the high complexity condition, the mixture of heuristics increased discrimination sharply (90%), making the two sequential heuristics the most useful where discrimination between alternatives was concerned. The difference in performance between the two, however, was very different in favor of *TTEB then TTB*. It was already found that TTEB alone fitted actual choices much better than TTB alone, which makes this result predictable. However, the sequential combination of the two, starting with TTEB increased discrimination to 89%. In terms of performance, there was a marginally significant decrease in overall performance (from 63% to 51%; z = 2.01; p = .04).

In sum, the results of how well the different choice models and heuristics predicted actual choices show that under low complexity, when there is cognitive/affective equivalence all models performed very similarly and are redundant. When there was cognitive/affective ambivalence however, only WADD and the heuristics that use integral affective responses are able to predict choices significantly better than random. TTB does not seem to fit actual choices under cognitive affective ambivalence. However, increasing complexity and complexity change the results considerably. First, WADD is unable to predict any kind of choice. Second, only the models that contain affective reactions (i.e., *TTEB* alone and *TTEB then TTB*) perform significantly and consistently better than random choice under both cognitive/affective equivalence and ambivalence.

## Discussion

This paper advances knowledge about how people balance affect and deliberation to determine their preferences within the bounded rationality framework. In particular, we focused on the problem of conflicting preferences between cognition and affect. We manipulated decision complexity through cognitive load in order to alter the decision environment not only through information processing demands but also by providing conditions for cognitive/affective ambivalence to appear. We found such ambivalence even in the simple, low complexity condition. Through our model comparison performance test, we found that reliance on the affective TTEB heuristic is enhanced by complexity and cognitive/affective ambivalence. However, as the informational environment grows in complexity, TTEB loses discrimination capacity, which seems to be complemented by the use of TTB wherever TTEB cannot discriminate. This finding suggests a novel view, in that decision makers may use a sequential, blended affective/cognitive heuristic processing where affect potentially precedes deliberation in decision tasks, particularly where discrimination between alternatives is increasingly difficult. Note that in the design of this research there were no time constraints forcing decisions on fast and automatic modes. Decision makers had the time and information to employ any strategy.

Can *TTEB* and *TTEB then TTB* be considered heuristics? Following Gigerenzer’s [4] conceptualization of heuristics, TTEB describes a processing strategy based on the informational value of integral affective responses [6]. Our experimental results suggest that TTEB is used when complexity and/or cognitive/affective ambivalence increases. That is, the decision environment where TTEB works better is one where information-processing demands are high and where affect and deliberation points in different directions. Interestingly, complex tasks may decrease TTEB’s discrimination capacity, which is then complemented by the use of TTB. With these results, we describe both the process and the cognitive architecture under which such processes work, providing an explanation of the ecological validity of *TTEB* and *TTEB then TTB*.

These findings also inform how dual systems of thought interact. The presence of cognitive/affective ambivalence suggests a conflict between System I and System II, or hot/cold modes, because the emotional component of System I (automatic) influences preferences in one direction, and System II (deliberate) does so in another direction. Lowenstein et al.´s [14] model of the interplay between affect and deliberation, based on a robust body of literature, emphasizes that conflict between the two systems is somehow solved by reliance on one of the two. Reliance on affect is expected to be increased by loss of willpower, reduced self-control or environmental conditions such as complexity. Our findings however, show that sequential heuristic processing solves such system conflicts. Hence, there is no tradeoff between affect and deliberation. Instead, there seems to be an adaptive, heuristic mechanism that entails the complementary roles of affective and cognitive heuristics. A salient factor that contributes to such "cooperation" is the problem of discrimination ability. Our findings suggest that, in this sequential blended heuristic processing, affect precedes cognition. That is, preferences are first determined by "how do I feel about it" [18] and where no discrimination is possible, TTB or a lexicographic procedure is used complementarily. We suggest that this highlights the informational worth of integral affective responses as they allow the DM to express the overt and covert value of alternatives. TTB helps emotions to inform preferences as much as possible by closing the gaps. Moreover, this notion of mixed heuristic processing is consistent with the idea of variable and reciprocal cue validities that are reassessed through the decision process in order to achieve a coherent representation of the task [59], as well as with the notion of blended decision strategies [38]. Thus, affect and cognition would act reciprocally to achieve the same objective.

This research contributes to understanding how affect is processed when deciding by "liking" [60]. It is known—based on the notions of how-do-I-feel-about-it [40], the affect heuristic [20] [32], and affect referral [61]- that affect guides choice, and, given its automatic nature, it is very likely that it usually precedes or overrides attempts at multiattribute calculations (See a review in Frederick, [61]). Much of such research has been devoted to determine the virtues and problems of reliance on affect as if it were competing with cognitive choice algorithms. For instance, reliance on affect is convenient if affective responses are aligned to subsequent enjoyment. Affective impressions may also be convenient because they may provide more accurate evaluations than cognitive processes when assessing choice alternatives. The present work offers, instead, a description of the way affect and cognition work together to help the decision maker overcome ambivalence and cognitive demands, highlighting the ecological rationality of such complementarity. Affective responses are malleable and context dependent [62] which contributes to avoiding the arguably unnecessary quest of coherence and rationality in decision-making processes [63]. Our research brings us a step closer to understanding the dance of affect and reason within the incoherent principles of behavior that humans use in order to be functional decision makers in an imperfect world.

This research also constitutes a step forward in the integration of affect into the debate about adaptive decision making and strategy selection [2]. Is affect-based decision making part of a multi-mechanism model or is it a single mechanism that interacts with other cognitive strategies? Much more research would be necessary to answer that question, adding other variables such as response times and neural activity, as well other methods of model comparison. However, the present behavioral results based on decisional outcomes suggest that affect-based heuristics may blend with cognitive ones within a multi-mechanism approach, whereby the way strategies are blended is also part of the decision maker toolbox. Another ensuing question is whether the effect of the number of alternatives with respect to the number of attributes differentially influences strategy selection and reliance on affect by virtue of triggering distinguishable cognitive process and working memory load.

## Supporting information

S1 Table Alternatives and attributes by treatment. (DOCX)

S1 Fig Self-assessment manikin scales for pleasure, arousal, and dominance. (PDF)

S1 Data Data. (XLSX)
