# Supplementary material for: The complementary role of affect-based and cognitive heuristics to make decisions under conditions of ambivalence and complexity
Source: PLoS One. 2018 Nov 9;13(11):e0206724. doi: 10.1371/journal.pone.0206724 (PMC6226170; doi:10.1371/journal.pone.0206724)
Supplement: S1 Table — (DOCX) [file pone.0206724.s001.docx]

| S1 Table. Alternatives and attributes by treatment. | | | | | | | |
| --- | --- | --- | --- | --- | --- | --- | --- |
|  |  |  |  |  |  |  |  |
| **Low complexity** | | |  |  |  |  |  |
| Computers | | | | | |  |  |
|  | a. | **Screen size** |  | b. | **Screen size** |  |  |
|  |  | 17 inches |  |  | 14 inches |  |  |
|  |  | **Memory** |  |  | **Memory** |  |  |
|  |  | 2GB |  |  | 1GB |  |  |
| Cars | |  |  |  |  |  |  |
|  | a. | **Engine** |  | b. | **Engine** |  |  |
|  |  | 1700 cc |  |  | 1200 cc |  |  |
|  |  | **Warranty** |  |  | **Warranty** |  |  |
|  |  | 12 months |  |  | 19 months |  |  |
|    \| Restaurants \| \| --- \| |  |  |  |  |  |  |  |
|  | a. | **Waiting time** |  | b. | **Waiting time** |  |  |
|  |  | 5 minutes |  |  | 20 minutes |  |  |
|  |  | **Ranking** |  |  | **Ranking** |  |  |
|  |  | 32 |  |  | 19 |  |  |
| Cell phones | | |  |  |  |  |  |
|  | a. | **Battery duration (talking)** |  | b. | **Battery duration (talking)** |  |  |
|  |  | 10 hours |  |  | 4.5 hours |  |  |
|  |  | **Internal memory** |  |  | **Internal memory** |  |  |
|  |  | 4GB |  |  | 8GB |  |  |
| Digital camera | | |  |  |  |  |  |
|  | a. | **Photo definition** |  | b. | **Photo definition** |  |  |
|  |  | 5 megapixels |  |  | 9 megapixels |  |  |
|  |  | **Screen size** |  |  | **Screen size** |  |  |
|  |  | 4.2 inches |  |  | 2.8 inches |  |  |

| **High complexity** | | |  |  |  |  |  |  |  |  |  |  |
| --- | --- | --- | --- | --- | --- | --- | --- | --- | --- | --- | --- | --- |
|  |  |  |  |  |  |  |  |  |  |  |  |  |
| Computers | | | | | | | | | | | |  |
|  | a. | **Screen size** |  | b. | **Screen size** |  | c. | **Screen size** |  | d. | **Screen size** |  |
|  |  | 15 inches |  |  | 14 inches |  |  | 17 inches |  |  | 12 inches |  |
|  |  | **Memory** |  |  | **Memory** |  |  | **Memory** |  |  | **Memory** |  |
|  |  | 2GB |  |  | 1.5GB |  |  | 1GB |  |  | 512KB |  |
|  |  | **Hard drive** |  |  | **Hard drive** |  |  | **Hard drive** |  |  | **Hard drive** |  |
|  |  | 0.6TB |  |  | 1.2TB |  |  | 0.8TB |  |  | 1TB |  |
|  |  | **Battery duration** |  |  | **Battery duration** |  |  | **Battery duration** |  |  | **Battery duration** |  |
|  |  | 3 hours |  |  | 2 hours |  |  | 5 hours |  |  | 6 hours |  |
| Cars | | | | | | | | | | | |  |
|  | a. | **Engine** |  | b. | **Engine** |  | c. | **Engine** |  | d. | **Engine** |  |
|  |  | 1500 cc |  |  | 1700 cc |  |  | 1200 cc |  |  | 900 cc |  |
|  |  | **Warranty** |  |  | **Warranty** |  |  | **Warranty** |  |  | **Warranty** |  |
|  |  | 12 months |  |  | 6 months |  |  | 15 months |  |  | 19 months |  |
|  |  | **Fuel tank size** |  |  | **Fuel tank size** |  |  | **Fuel tank size** |  |  | **Fuel tank size** |  |
|  |  | 5 gallons |  |  | 8 gallons |  |  | 15 gallons |  |  | 11 gallons |  |
|  |  | **Acceleration 0-60 miles/h** |  |  | **Acceleration 0-60 miles/h** |  |  | **Acceleration 0-60 miles/h** |  |  | **Acceleration 0-60 miles/h** |  |
|  |  | 10 seconds |  |  | 15 seconds |  |  | 35 seconds |  |  | 25 seconds |  |
|    \| Restaurants \| \| --- \| | | | | | | | | | | | |  |
|  | a. | **Waiting time** |  | b. | **Waiting time** |  | c. | **Waiting time** |  | d. | **Waiting time** |  |
|  |  | 5 minutes |  |  | 10 minutes |  |  | 20 minutes |  |  | 30 minutes |  |
|  |  | **Average price per meal** |  |  | **Average price per meal** |  |  | **Average price per meal** |  |  | **Average price per meal** |  |
|  |  | $ 17 |  |  | $ 16 |  |  | $ 15 |  |  | $ 14 |  |
|  |  | **Ranking** |  |  | **Ranking** |  |  | **Ranking** |  |  | **Ranking** |  |
|  |  | 32 |  |  | 50 |  |  | 19 |  |  | 24 |  |
|  |  | **Commute time from home** |  |  | **Commute time from home** |  |  | **Commute time from home** |  |  | **Commute time from home** |  |
|  |  | 20 minutes |  |  | 10 minutes |  |  | 40 minutes |  |  | 30 minutes |  |
| Cell phones |  |  |  |  |  |  |  |  |  |  |  |  |
|  | a. | **Battery duration (talking)** |  | b. | **Battery duration (talking)** |  | c. | **Battery duration (talking)** |  | d. | **Battery duration (talking)** |  |
|  |  | 10 hours |  |  | 7 hours |  |  | 4.5 hours |  |  | 3 hours |  |
|  |  | **Time to recharge** |  |  | **Time to recharge** |  |  | **Time to recharge** |  |  | **Time to recharge** |  |
|  |  | 6 hours |  |  | 7.3 hours |  |  | 3 hours |  |  | 4.5 hours |  |
|  |  | **Internal memory** |  |  | **Internal memory** |  |  | **Internal memory** |  |  | **Internal memory** |  |
|  |  | 6GB |  |  | 8GB |  |  | 2GB |  |  | 4GB |  |
|  |  | **Processor´s speed** |  |  | **Processor´s speed** |  |  | **Processor´s speed** |  |  | **Processor´s speed** |  |
|  |  | 0.5 Hz |  |  | 0.9 Hz |  |  | 1.5 Hz |  |  | 2.2 Hz |  |
|    \| Digital cameras \| \| --- \| |  |  |  |  |  |  |  |  |  |  |  |  |
|  | a. | **Photo definition** |  | b. | **Photo definition** |  | c. | **Photo definition** |  | d. | **Photo definition** |  |
|  |  | 7 megapixels |  |  | 5 megapixels |  |  | 9 megapixels |  |  | 3 megapixels |  |
|  |  | **Zoom** |  |  | **Zoom** |  |  | **Zoom** |  |  | **Zoom** |  |
|  |  | 5x optic |  |  | 8x optic |  |  | 3x optic |  |  | 6x optic |  |
|  |  | **Peso** |  |  | **Peso** |  |  | **Peso** |  |  | **Peso** |  |
|  |  | 150 grams |  |  | 90 grams |  |  | 65 grams |  |  | 50 grams |  |
|  |  | **Screen size** |  |  | **Screen size** |  |  | **Screen size** |  |  | **Screen size** |  |
|  |  | 4.2 inches |  |  | 3.6 inches |  |  | 2.8 inches |  |  | 2.1 inches |  |
|  |  |  |  |  |  |  |  |  |  |  |  |  |
