# Supplementary material for: The complementary role of affect-based and cognitive heuristics to make decisions under conditions of ambivalence and complexity
Source: PLoS One. 2018 Nov 9;13(11):e0206724. doi: 10.1371/journal.pone.0206724 (PMC6226170; doi:10.1371/journal.pone.0206724)
Supplement: S1 Fig — (PDF) [file pone.0206724.s002.pdf]

S2 Fig. Self-Assessment Manikin Scales for Pleasure, Arousal, and Dominance

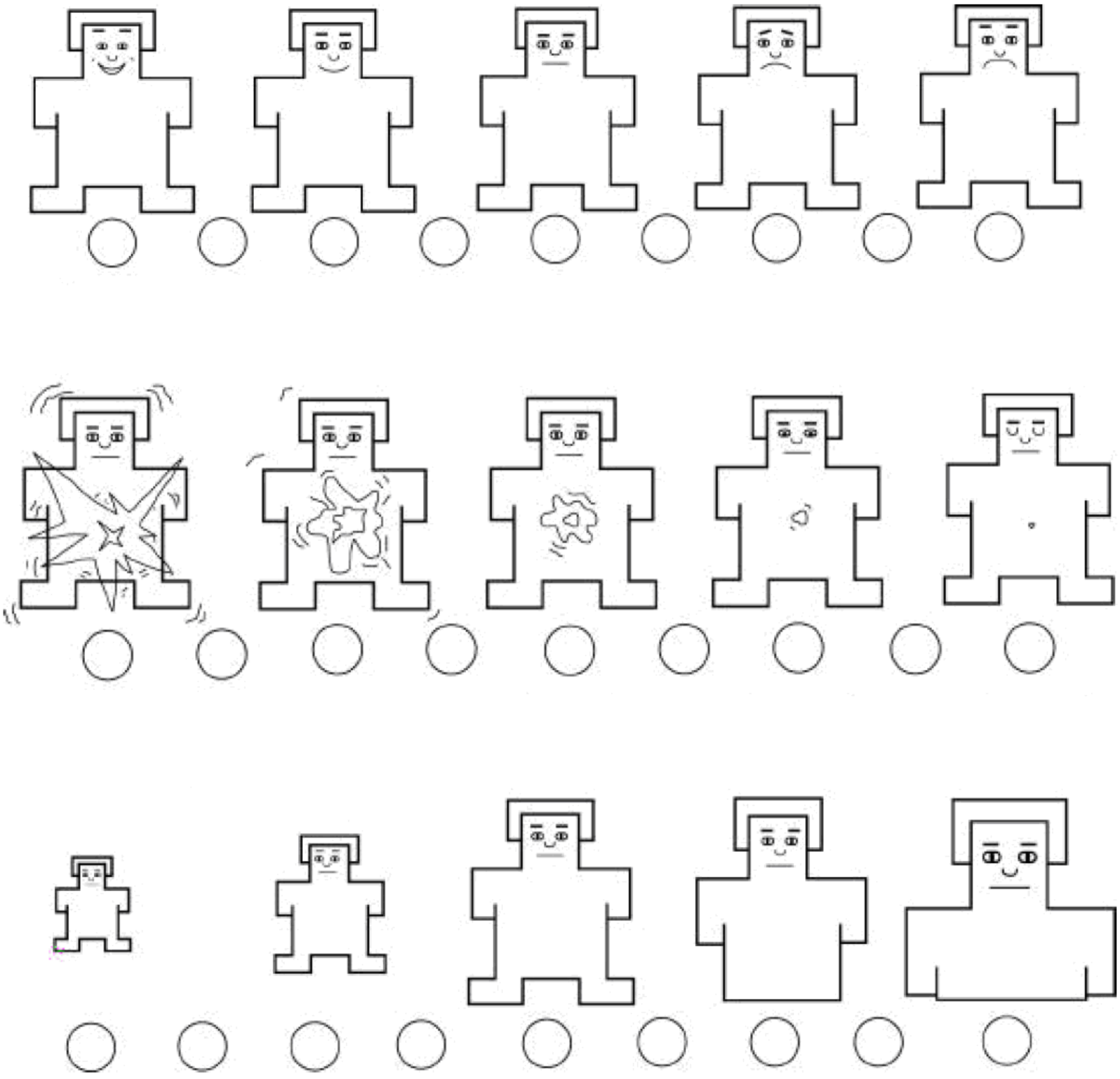

Copied from Bradley, M. M. and Lang, P. J. (1994). Measuring emotion: The self-assessment manikin and the semantic differential. Journal of Behavioral Theory and Experimental Psychiatry, 25 (1), 49-59
